# In vivo monitoring of the therapeutic efficacy of a CXCR1/2 inhibitor with 18F-FDG PET/CT imaging in experimental head and neck carcinoma: A feasibility study

**DOI:** 10.1016/j.bbrep.2021.101098

**Published:** 2021-08-12

**Authors:** Christopher Montemagno, Benjamin Serrano, Jérôme Durivault, Valérie Nataf, François Mocquot, Régis Amblard, Valérie Vial, Cyril Ronco, Rachid Benhida, Maeva Dufies, Marc Faraggi, Gilles Pagès

**Affiliations:** a Département de Biologie Médicale, Centre Scientifique de Monaco, Monaco; b Medical Physics Department, Centre Hospitalier Princesse Grace, Monaco, Monaco; c Nuclear Medicine Department, Centre Hospitalier Princesse Grace, Monaco, Monaco; d Institute for Research on Cancer and Aging of Nice, Université Cote D’Azur, CNRS UMR 7284, INSERM U1081, Centre Antoine Lacassagne, 06200, Nice, France; e Université Côte D'Azur, CNRS, Institut de Chimie de Nice UMR 7272, 06108, Nice, France

**Keywords:** CXCR1/2, HNSCCs, Chemical inhibitor, ^18^F-FDG, PET/CT imaging

## Abstract

The chemokine receptors CXCR1/2 play a key role in the aggressiveness of several types of cancers including head and neck squamous cell carcinomas (HNSCCs). In HNSCCs, CXCR1/2 signaling promotes cell proliferation and angiogenesis leading to tumor growth and metastasis. The competitive inhibitor of CXCR1/2, C29, inhibits the growth of experimental HNSCCs in mice. However, a non-invasive tool to monitor treatment response is essential to implement the use of C29 in clinical practices. ^18^F-FDG PET/CT is a gold-standard tool for the staging and the post-therapy follow-up of HNSCCs patients. Our study aimed to perform the first i*n vivo* monitoring of C29 efficacy by non-invasive ^18^F-FDG PET/CT imaging. Mice bearing experimental HNSCCs (CAL33) were injected with ^18^F-FDG (T0) and thereafter treated (n = 7 mice, 9 tumors, 50 mg/kg by gavage) or not (n = 7 mice, 10 tumors) with C29 for 4 consecutive days. Final ^18^F-FDG-tumor uptake was determined at day 4 (TF). The average relative change (TF-T0) in ^18^F-FDG tumor uptake was +25.85 ± 10.93 % in the control group *vs* −5.72 ± 10.07 % in the C29-treated group (p < 0.01). These results were consistent with the decrease of the tumor burden and with the decrease of tumor proliferating Ki67+ cells. These results paved the way for the use of ^18^F-FDG to monitor tumor response following C29 treatment.

## Highlights

• CXCR1/2 inhibitors represent a new class of anti-cancer drugs.
• CXCR1/2 inhibition delays HNSCCs tumor growth.
• ^18^F-FDG allows early monitoring of CXCR1/2 inhibitors efficacy.

## 1 Introduction

Most head and neck cancers are derived from the mucosal epithelium in the oral cavity, pharynx and larynx and are known as head and neck squamous cell carcinoma (HNSCC). HNSCC is the sixth most common cancer worldwide with 890,000 new cases and 450,000 deaths in 2018 [1,2]. Its incidence is expected to increase by 30% by 2030 [3]. This dramatic increase is mainly due to alcohol and tobacco habits, two drivers in HNSCCs etiology [4], but also to oncogenic viruses such as human papillomavirus (HPV) and Epstein-Barr virus (EBV) [4]. HNSCCs are aggressive tumors and up to 50 % of patients will develop loco-regional relapse or metastases within 2 years [[5], [6], [7]]. Early stage HNSCC can be treated with single-modality therapy with surgical resection and radiotherapy (RT). A combination of surgery, chemotherapy and/or radiotherapy is indicated for patients with regionally advanced cancers; whereas surgery, RT and a broad range of systemic therapies are indicated for metastatic cancers [8]. In the recent years, the advent of immunotherapy has open new opportunities for recurrent/metastatic (R/M) HNSCC. The Food and Drug Administration (FDA) recently approved nivolumab (anti-programmed cell death protein 1 (PD1) antibody) and pembrolizumab (anti programmed cell death protein ligand 1 (PD-L1) as second line treatments of R/M HNSCC patients and pembrolizumab in first-line in PD-L1 (combined positive score ≥ 1%) tumor-positive patients [9,10]. Unfortunately, only 30% of patients respond to such therapies [11]. The development of new drugs is an urgent need for the management of patients with R/M HNSCCs ineligible to such therapies. Up to 90% of HNSCC overexpress epidermal growth factor receptor (EGFR) and/or angiogenic factors such as vascular endothelial growth factor (VEGF), which drives hypervascularization and metastatic dissemination. However, *anti*-EGFR are used only at very late-stage, and fatal hemorrhages were described following *anti*-VEGF therapies. Therefore, anti-angiogenic drugs used alone or in combination with chemotherapy in R/M HNSCC failed to demonstrate a significant increase of overall survival (OS) [12,13]. Angiogenesis and inflammation are two interconnected hallmarks of cancer. ELR+CXCL cytokines (CXCL1, 2, 3, 5, 6, 7 and 8) and their G-protein coupled receptors (CXCR1 and CXCR2) have recently emerged as relevant therapeutic targets for their roles in these processes [[14], [15], [16]]. ELR+CXCL cytokines are overexpressed in HNSCCs and correlated to poor survival [17]. CXCL5 and CXCL8 correlated to HNSCCs aggressiveness [18,19]. Moreover, at least CXCL8 is induced in response to radiotherapy [20]. These observations favors the relevance of targeting the ELR+CXCL/CXCR axis for HNSCC. We previously reported the use of a new pharmacological inhibitor of CXCR1/2, C29. This compound, namely 1-(3-chlorophenyl)-3-(6-nitrobenzo[d]thiazol-2-yl)urea, belongs to the diarylurea molecular class and presents a 2-aminobenzothiazolyl moiety. It is the lead compound of a series of molecules developed by rational design from scaffold hopping and dockings studies on the structures of CXCR1 and CXCR2 receptors. C29 exerts high anti-proliferative activities in a panel of cancer cells, including naive and radiotherapy-resistant HNSCC cells. C29 also reduced the growth of experimental HNSCC in mice [17].

An accurate evaluation of the tumor's therapeutic response is essential for the management of patients with HNSCC. ^18^F-fluorodeoxy-d-glucose positron emission tomography-computed tomography (^18^F-FDG PET-CT) allows pre-treatment staging, treatment response assessment and post-therapy follow-up in HNSCC [21,22]. ^18^F-FDG PET/CT is more performant as compared to other imaging modalities to detect relapses of loco-regional diseases and distant lesions [21]. In this feasibility study, we aimed at investigating the relevance of ^18^F-FDG PET/CT for the early monitoring of C29 efficacy in experimental HNSCC.

## 2 Materials and methods

### 2.1 Tumor model and treatment

**Fig. 1:**
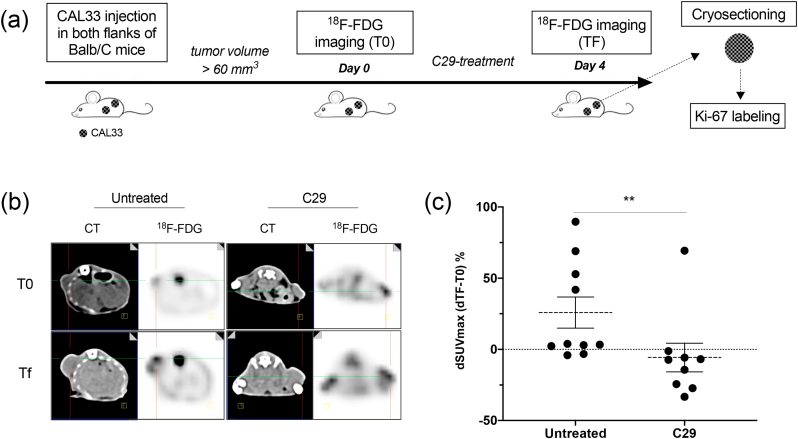
***In vivo* monitoring of C29 efficacy by**^**18**^**F-FDG PET/CT. (a)**. Flow chart of the study. **(b)**. Representative CT images (left) and ^18^F-FDG PET images (right) of tumors from control (left panel) or C29-treated (right panel) mice performed at baseline (T0) and 4 days after treatment (TF). **(c)**. Evolution of ^18^F-FDG tumor uptake in CAL33 tumors between TF and T0. Relative changes between TF and T0 in normalized tumor uptake was plotted. **p < 0.01 vs untreated.

All procedures were performed in accordance with the institutional guidelines and approved by the animal care and use committee of Monaco (Veterinary service and direction of sanitary and social action of Monaco, Dr. H. Raps, PEA n°57). For this study, one million CAL33 cells (RRID:CVCL_1108, human tongue squamous cell carcinoma) were subcutaneously injected in the two flanks of fourteen 6-weeks-old female BALB/cjRj Nude mice (Janvier Labs). Tumors’ volumes were evaluated with a caliper. When at least one of the two tumors reached around 100 mm^3^ (which corresponds to 60 mm^3^ on CT-based measurement), ^18^F-FDG PET/CT acquisitions were performed (T0). Following this acquisition, mice were treated (n = 7 mice, 9 tumors) or not (n = 7 mice, 10 tumors) with C29 (50 mg/kg) by gavage for 4 consecutive days (Fig. 1 (a)). Then, a final ^18^F-FDG PET/CT imaging was performed immediately after 4 days of treatment (TF). The 4-days period was therefore chosen for the early monitoring by ^18^F-FDG PET/CT. Following the last acquisition, mice were euthanized, and tumors were weighted and kept for *ex vivo* analyses (Ki67 labeling).

### 2.2 Image acquisition and image processing

CT-scans were performed during the ^18^F-FDG PET/CT using a Biograph Vision 600 Edge (Siemens) and were realized with a 512x512 matrix, 128 slices/rotation and reconstructed in a 0.5 mm every 0.3 mm with a 0.6 pitch. The FOV was around 60 mm and the final CT voxel size was 0.12x0.12x0.5 mm^3^. An abdominal view and an I50f medium Sharp ASA filter were used with an iteration software (ADMIRE) for a tube voltage peak of 70 kVp and a fixed 415 effective mAs. Mice were injected via the tail vein with 13.20 **±** 3.80 MBq of ^18^F-FDG. PET/CT acquisitions were performed 45 min after injection. Imaging was acquired during 15 min using one step Time of Flight (TOF), attenuation and scatter correction and a 880x880 matrix. The images were reconstructed with 5 subsets and 30 iterations using the TrueX+TOF (ultraHD-PET) algorithm and gaussian filter (FWHM = 1 mm) and a zoom = 3. The PET voxel size was 0.275x0.275x1.65 mm^3^. The minimal tumor volume studied was 60 mm^3^. Images were quantified after correction for decay and normalization to the injected dose. Normalized tumor ^18^F-FDG uptake was computed as the ratio of the tumor maximum Standardized Uptake Value (SUVmax) divided by the mean Liver Standardized Uptake Value (SUVmean). Normalized tumor ^18^F-FDG uptake = Tumor SUVmax /Liver SUVmean.

### 2.3 Ex vivo analyses: immunofluorescence

Immediately after the last acquisition, tumors were harvested, weighted and embedded in OCT for cryosectioning. Tumor sections (5-μm cryostat sections) were ﬁxed in 4 % paraformaldehyde for 10 min at room temperature and blocked in 1 % horse serum in Tris-buffered saline (TBS) for 1 h. Sections were then incubated overnight at 4 °C with *anti*-Ki67 (ab16667, 1:500; Abcam) antibody. Preparations were mounted and analyzed with a Leica microscope (Leica DMI4000B) and counted at a 40x magnification.

### 2.4 Statistical analysis

Data are presented as the mean ± standard error of the mean (SEM). Statistical differences inside a same group (paired values, CT-based and caliper-based volumes, initial ^18^F-FDG uptake) at different times of experimentation were compared using non-parametric Wilcoxon test. Inter-group analyses (unpaired values, delta tumor volumes (TF-T0, delta ^18^F-FDG uptake (TF-T0) were compared using non-parametric Mann-Whitney test. p < 0.05 was considered as significant.

## 3 Results

### 3.1 ^18^F-FDG PET/CT accuracy for monitoring therapeutic response

The accuracy of three independent methods to evaluate the therapeutic effects of C29 was evaluated. At baseline, no significant difference was found in experimental tumors of the two randomized groups in either caliper-based and CT-based tumor volume measurements or tumor ^18^F-FDG-uptake (Table 1). While all theses parameters significantly increased between T0 and TF in untreated control mice, no difference was found in treated mice. Transversal fused ^18^F-FDG PET/CT sections of tumors from untreated or C29-treated mice at baseline (T0) and TF are presented in Fig. 1(b) and in Fig. S1. The average relative change in normalized ^18^F-FDG uptake was 25.85 ± 10.93% in tumors of the control group *vs* −5.72 ± 10.07% in tumors from C29-treated mice (p = 0.004, Table 1, Fig. 1(c)). Accordingly, the weight of tumors from C29-treated mice was significantly smaller (Table 1).

**Table 1:** Summary of the different parameters evaluated throughout the study.

		Untreated		C29-treated		p-value between Untreated/C29-treated
	Number of tumors	10		9		–
**CT-based Tumor volume (mm**^**3**^**)**	**Initial (T0)**	95 ± 8	p = 0.002	107 ± 15	NS	NS
	**Final (TF)**	212 ± 37**		146 ± 26		**0.047**
**delta CT-based volume (% of change)**	**[Final (TF) - Initial (T0)] /Initial (T0)**	123.37 ± 31.76%		17.93 ± 10.63%		**0.0032**
**Caliper-based Tumor volume (mm**^**3**^**)**	**Initial (T0)**	155 ± 13	p = 0.008	161 ± 11	NS	NS
	**Final (TF)**	320 ± 34**		218 ± 23		**0.0335**
**delta caliper-based volume (% of change)**	**[Final (TF) - Initial (T0)] /Initial (T0)**	103.88 ± 21.38%		37.15 ± 13.83%		**0.0350**
^**18**^**F-FDG uptake (Normalized SUVmax)**	**Initial (T0)**	5.61 ± 0.44	p = 0.033	6.19 ± 1.26	NS	NS
	**Final (TF)**	7.16 ± 0.90*		5.73 ± 1.00		NS
**delta**^**18**^**F-FDG uptake (% of change)**	**[Final (TF) - Initial (T0)] /Initial (T0)**	25.85 ± 10.93%		−5.72 ± 10.07%		**0.004**
**Tumor weight (mg)**	**Final (TF)**	189 ± 11		139 ± 12		**0.0106**

These results suggest that relative change in ^18^F-FDG-uptake is a valuable method to monitor the efficacy of C29 in inhibiting the growth of experimental HNSCC in mice as soon as 4 days.

### 3.2 C29 treatment decreases the number of CAL33 proliferative cells

**Fig. 2:**
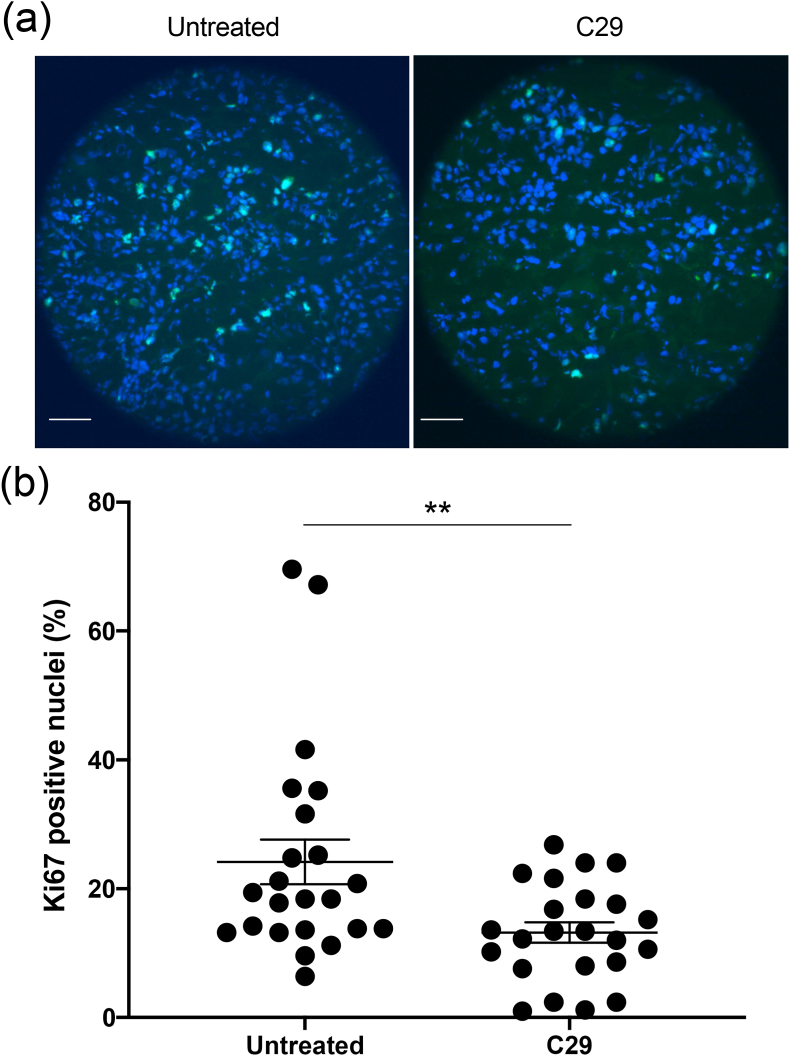
**Ki-67 labeling of CAL33 tumor sections**. (a) Representative images of Ki67 immunolabeling (green) and Hoechst33342 nuclear DNA counterstaining (blue). Scale bar: 50 μm. (b) Quantification of Ki67-positive cells in CAL33 tumors. **p < 0.01 vs untreated mice.

The expression of Ki67 protein (Ki67), which correlates with the proliferative activity of intrinsic cell populations in malignant tumors, was determined *ex vivo*. C29 treatment is associated with a 45% decrease in the number of Ki67-positive nuclei as compared to tumors from untreated mice (13.19 ± 1.58% vs 24.17 ± 3.46%, P < 0.01, Fig. 2(a and b), Fig. S2).

## 4 Discussion

After the demonstration of the anti-tumor effect C29, we aimed to evaluate the efficacy of a CXCR1/2 inhibitor in preclinical model of HNSCC as early as possible after the onset of treatment using ^18^F-FDG PET-CT. Such evaluation represents a potent asset for further implementation in the clinic of our patented compound (WO 2020/079,184).

The advent of new classes of anti-cancer drugs such as immunotherapies, with the use of *anti*-PD-L1 and *anti*-PD1 antibodies, have considerably improve the outcome of patients with solid tumors, including R/M HNSCC [9]. Nevertheless, few patients are eligible to such therapies and the development of new class of drugs is an urgent need. We previously reported that the ELR+CXCL-CXRC1/2 axis is associated with a poor outcome in HNSCC [17]. This axis is involved in several hallmarks of cancer including angiogenesis, immune tolerance, and tumor cell proliferation. Indeed, we believed that a CXCR1/2 pharmacological inhibitor could constitute a “blasting missile” for R/M solid tumors. We demonstrated that C29 inhibited the proliferation of cisplatin resistant human HNSCC cells and reduced the burden of experimental HNSCC.

The development of new anti-cancer agents, such as C29, or the monitoring of approved therapies require non-invasive methods to evaluate their efficacy. ^18^F-FDG PET-CT is commonly used to assess treatment response, since it identifies viable tumor within residual masses, overcoming the limitations of morphological imaging modalities such as CT [21]. Early ^18^F-FDG PET-CT, one week post-therapy, is predictive of relapse and prognosis in HNSCC patients treated with chemo- or radiotherapy [[23], [24], [25]]. Several preclinical and clinical studies have investigated the relevance of ^18^F-FDG PET/CT for treatment monitoring in different cancers. The advent of ^18^F-FDG PET has considerably improved the evaluation of treatment efficacy following chemo- and radio-therapy in HNSCC patients [26,27]. ^18^F-FDG PET is now commonly used to assess treatment response. It is a highly sensitive technique for the detection of R/M HNSCC, since it can identify persistent metabolically active tumor cells in residual masses [25].

In the current study, we showed that the variation (TF-T0) of ^18^F-FDG uptake significantly increased in untreated mice whereas such an increase was not observed in C29-treated mice. Individual variability of the injected dose is inevitable. Therefore, we chose to express the ^18^F-FDG tumor uptake as tumor SUVmax normalized to the mean liver uptake (SUVmean). Indeed, the hepatic uptake of ^18^F-FDG is homogeneous and important on rodents. Hence, the Tumor SUVmax/Liver SUVmean ratio allowed an accurate and reproducible internal standardization. We showed that 4 days of treatment were sufficient to observe a significant difference in the average (TF-T0) ^18^F-FDG uptake between untreated and treated groups. The growth rate of tumors generated with CAL33 cells was very short (doubling time of approximately 3 days). Thus, a period of 4 days was chosen to investigate the efficacy of C29 at early time points**.**

The results of the present study are consistent with our previous ones demonstrating the *ex-vivo* efficacy of the compound. Caliper- and CT-based volumes in C29-treated mice increased, while ^18^F-FDG uptake decreased suggesting a cytostatic effect of C29 at least during the very first days of treatment [[28], [29], [30]]. Nevertheless, these results illustrate the difficulties to interpret changes in tumor volume in clinical practices. Despite tumor volume increased to a lesser extent in C29-treated as compared to untreated mice, the stable or the slight decrease in ^18^F-FDG uptake appears more relevant for monitoring the therapeutic response. This limitation of tumor volume measurements with anatomic imaging has been reported for a long time in experimental tumors and patients treated by anti-angiogenic therapies [30,31]. A more important decrease of ^18^F-FDG-tumor uptake must be validated at longer time points. Nevertheless, as early as 4 days following C29 administration, a significant difference in mean relative changes in normalized ^18^F-FDG-tumor uptake was observed between treated and untreated mice. These results are consistent with the modifications of tumor volumes and with the decrease in the number of proliferative cells in tumors from C29-treated mice.

Following this feasibility study, further work will aim at investigating the predictive value of ^18^F-FDG uptake in C29-treated mice. Indeed, in the C29-treated group, one tumor did not respond to the treatment following 4 days of treatment. Longer time points should have determined if this tumor was an outlier for which C29 is inefficient or if a longer treatment was necessary. A longer period of follow-up is therefore needed to validate ^18^F-FDG as a predictive method to validate C29 efficacy.

The main limitation of this study was the use of a clinical PET system for which the resolution is not as high as dedicated small animals PET systems and may lead to partial volume effects. We chose to trigger PET/CT imaging when the tumor volume exceeded 60 mm^3^ to prevent this limitation. Moreover, partial volume effect might have reduced the ability to detect a decrease in ^18^F-FDG uptake in tumors of C29-treated mice if the tumor volume decrease. However, it could not explain the increase of ^18^F-FDG-tumor uptake in control mice.

## 5 Conclusion

The rapidity to assess C29 efficacy, in a context of “drug discovery” as well as the availability of ^18^F-FDG worldwide add valuable perspectives to our work. In a preclinical perspective, it will be possible to investigate the relevance of anti-cancer agents at early time points (from 4 days in this case). Moreover, in the context of the clinical transfer of C29, these preliminary results suggest that ^18^F-FDG PET/CT could be a valuable tool for monitoring treatment response in HNSCC.

## Author contributions

Conceptualization; M.F., G.P. Data curation; C.M., B.S., J.D.,V.N., F.M., R.A., V.V., C.R., R.B., M.D. Formal analysis; C.M., B.S., M.F. Funding acquisition; M.F., G.P. Investigation; C.M., B.S., J.D.,V.N., F.M., R.A., V.V., C.R., R.B., M.D. Methodology; C.M., B.S., V.N., M.F., G.P. Project administration; M.F., G.P. Resources; Software; B.S., C.M Supervision; M.F., G.P. Validation; M.F., G.P. Visualization; C.M., B.S., J.D., V.N., F.M., R.A., V.V., C.R., R.B., M.D., M.F., G.P. Writing - original draft; C.M., B.S., M.F., G.P.

## Declaration of competing interest

The authors declare that they have no known competing financial interests or personal relationships that could have appeared to influence the work reported in this paper.
