# Supplementary material for: In vivo monitoring of the therapeutic efficacy of a CXCR1/2 inhibitor with 18F-FDG PET/CT imaging in experimental head and neck carcinoma: A feasibility study
Source: Biochem Biophys Rep. 2021 Aug 12;27:101098. doi: 10.1016/j.bbrep.2021.101098 (PMC8374394; doi:10.1016/j.bbrep.2021.101098)
Supplement: Multimedia component 1 [file mmc1.docx]

*Supplementary data*


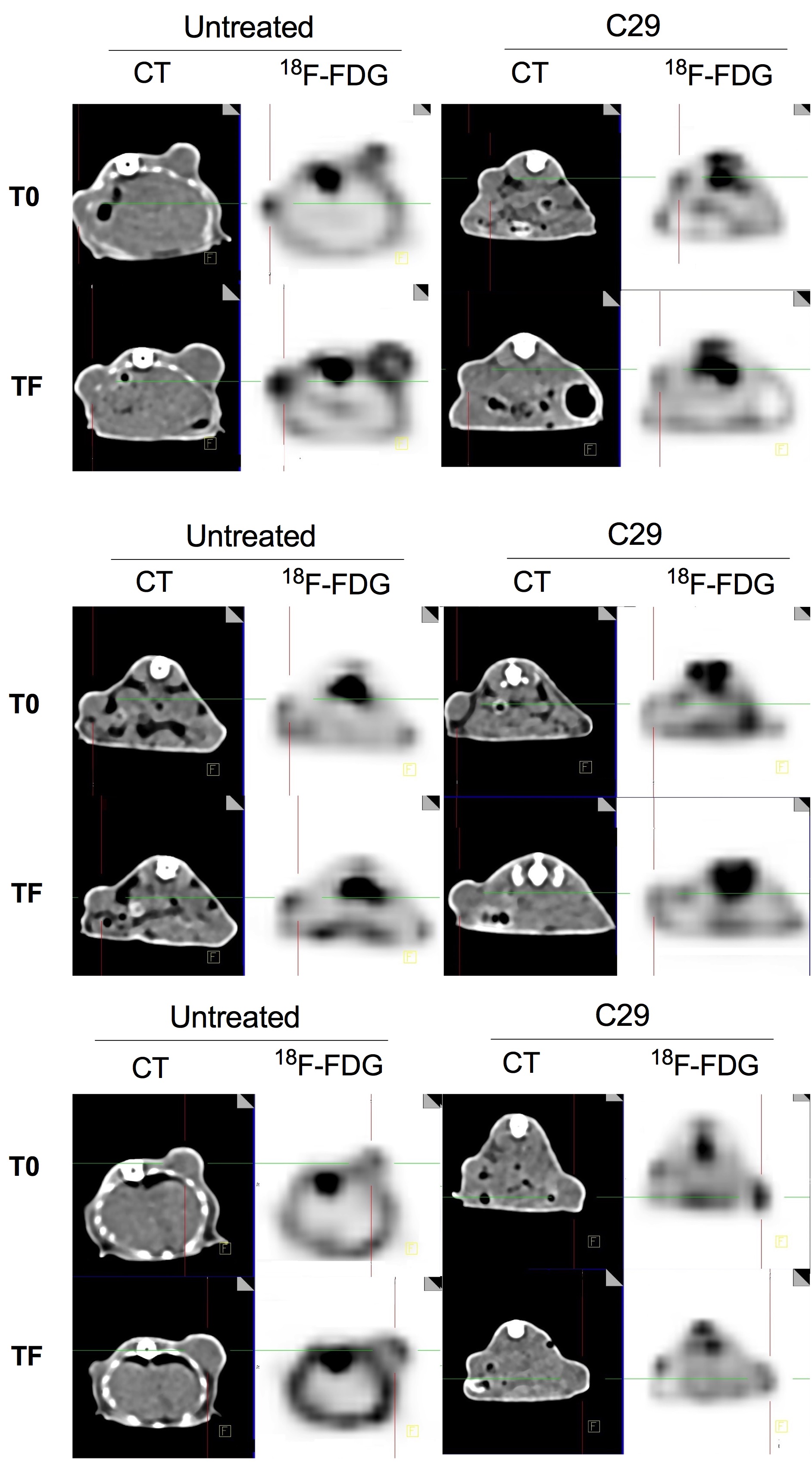


**Figure S1. Representative CT images (left) and ^18^F-FDG PET images (right) of tumors from 3 control (left panel) and 3 C29-treated (right panel) independant mice performed at baseline (T0) and 4 days after treatment (TF).**

**
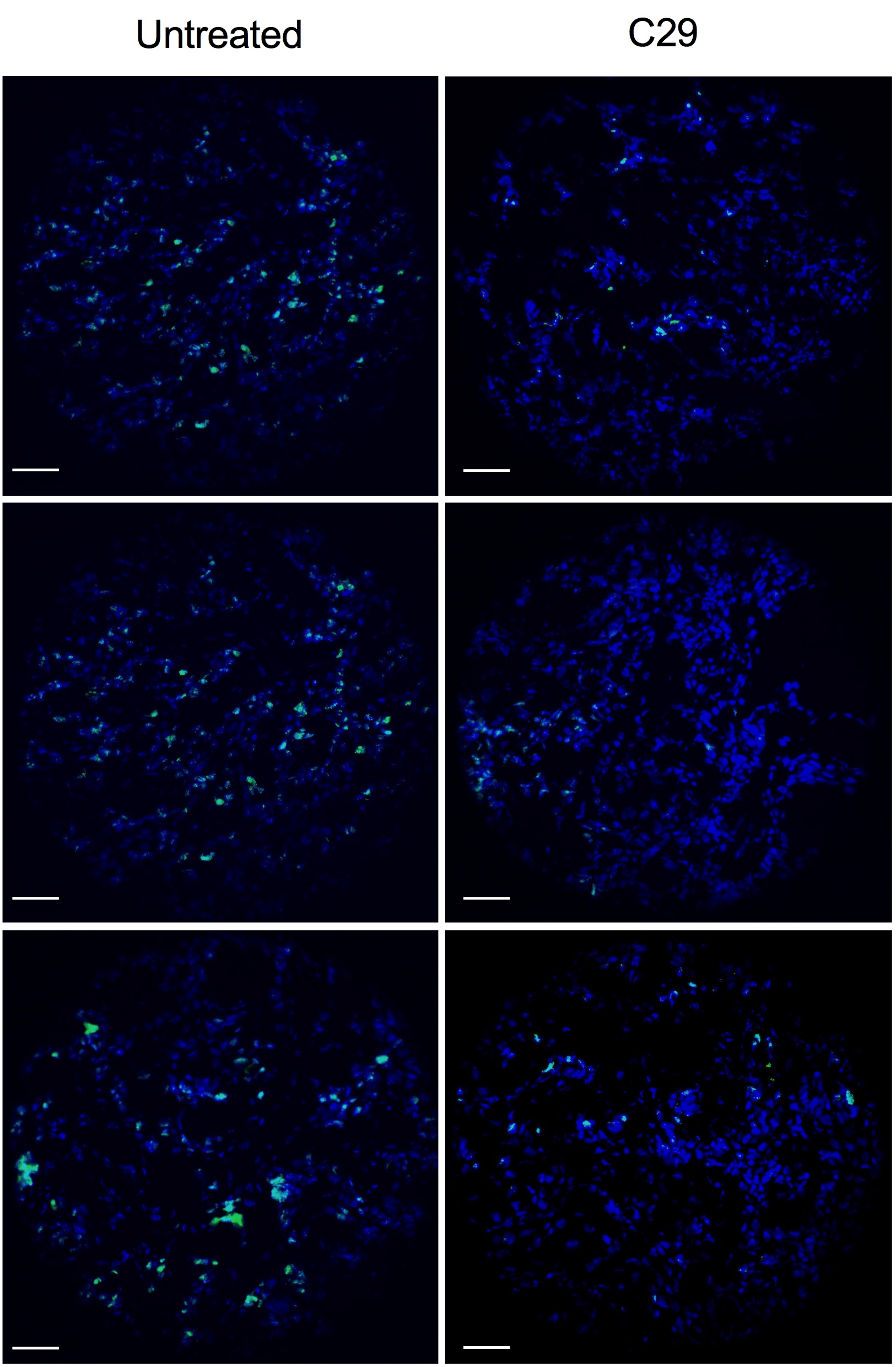
**

**Figure S2. Representative images of Ki67 immunolabeling (green) and Hoechst33342 nuclear DNA counterstaining (blue). Three independant tumors are represented. Scale bar: 50 μm**
